# The complete chloroplast genome of *Crataegus bretschneideri* Schneid. (Rosaceae)

**DOI:** 10.1080/23802359.2021.1994896

**Published:** 2021-11-03

**Authors:** Shuqi Zheng, Han Song, Ningguang Dong

**Affiliations:** a Beijing Academy of Forestry and Pomology Sciences, Beijing Academy of Agriculture and Forestry Sciences, Beijing, P.R. China; b Key Laboratory of Biology and Genetic Improvement of Horticultural Crops (North China), Ministry of Agriculture and Rural Affairs, Beijing, P.R. China; c College of Biological Sciences and Biotechnology, Beijing Forestry University, Beijing, P.R. China

**Keywords:** *Crataegus bretschneideri*, chloroplast genome sequence, phylogenetic analysis

## Abstract

*Crataegus bretschneideri* Schneid., with an unclear phylogenic position, is mainly distributed in northeast and inner mongolia area of China. In this study, the complete chloroplast genome sequence of *C. bretschneideri* was determined by using Illumina high-throughput sequencing method. The chloroplast genome was 159,607 bp in length and consisted of a large single-copy (LSC) region (87,601 bp), a small single-copy (SSC) region (19,312 bp), separated by a pair of inverted repeat (IRs: 26,347 bp, each) regions. It comprised a total of 114 unique genes, including 80 protein-coding genes, 30 tRNA genes, and 4 rRNA genes. Phylogenetic analysis based on complete chloroplast genomes indicated that *C. bretschneideri* was closely related to *C. marshallii* Eggl in the subfamily Maloideae. This complete chloroplast genome will provide valuable insight into evolution, molecular breeding, and phylogenetic analysis of *Crataegus* species.

The genus *Crataegus* (hawthorn), a member of the Rosaceae family, is widely distributed throughout temperate regions in the Northern Hemisphere including Eurasia and North America (Phipps et al. 1990; Christensen 1992; Du et al. 2019). Hawthorns are one of the most important processing and table fruits in China, owing to their nutrient-rich fruit and significant medicinal values (Özcan et al. 2005; Xu et al. 2016; Zheng et al. 2018). A total of 18 species and six varieties of *Crataegus* have been confirmed in China (Zhao and Feng 1996; Xin and Zhang 1997). *Crataegus bretschneideri* Schneid., originated from Changbaishan Massif of China, is mainly distributed in northeast and inner mongolia area of China (Zhao and Feng 1996). It is an important germplasm of *Crataegus* in China, with the characteristics of high yield, early-maturing and cold resistance. Its fruit is rich in nutrition, especially in natural red pigment, and has excellent processing properties.

*C. bretschneideri* is morphologically very similar to *C. pinnatifida* Bge., and Dai (2007) consider the former to be a variant of the latter species. Most of the morphological characters, including leaf color, leaf shape, leaf margin, fruit shape, peel color, etc, are similar between the two species. The most obvious differences between *C. bretschneideri* and *C. pinnatifida* are in the leaf blade lobes and seed number. Based on peroxidase isozymograms and inter-simple sequence repeat (ISSR) markers, some researchers suggest that *C. bretschneideri* is closely related to *C. pinnatifida* (Schneider 1906; Guo and Jiao 1995; Han et al. 2009). Specific locus amplified fragment sequencing revealed that *C. bretschneideri* was derived from the hybridization of *C. pinnatifida* with *C. maximowiczii* Schnieid (Du et al. 2019).

Chloroplast genomes are important sources for taxonomic classification and phylogenetic reconstruction of plant species (Dong et al. 2017; Liu et al. 2020; Wang et al. 2021). In order to clarify the phylogenetic position of *C. bretschneideri*, we reported the complete chloroplast genome based on Illumina sequencing data (GenBank accession number: MW963339), which would be helpful for evolution, phylogenetic analysis and molecular breeding.

The sample of *C. bretschneideri* was collected from the Hawthorn Germplasm Repository of Beijing Academy of Forestry and Pomology Sciences (39°97′N, 116°23′E) in Beijing, China. A specimen was deposited at the Herbarium of Beijing Academy of Forestry and Pomology Sciences (BAFPSH, http://www.lgs.baafs.net.cn/, Yuanyong Qi, bjlgsbgs@126.com) under the voucher number BJLGY-2020-SZ002. The total genomic DNA from leaves was extracted using a modified CTAB method (Li et al. 2013) and paired-end libraries were prepared with the NEBNext Ultra DNA Library Prep Kit. High-throughput sequencing was carried out using the HiSeq Xten PE150 System (Illumina, San Diego, CA, USA) with150bp pair-end reads. In all, 1.72 G raw reads were obtained, and after the quality-trimmed using the software CLC Genomics Workbench v7.5 (CLC bio, Aarhus, Denmark), 1.72 G qualified reads were assembled using SPAdes 3.6.1 (Kmer = 95) (Bankevich et al. 2012) to contigs. The contigs of chloroplast genome were selected with the BLAST program (Altschul et al. 1990), taking the closely related species *C. hupehensis* (MW201730) as a reference, and the selected contigs were assembled using Sequencher 4.10 (https://www.genecodes.com/) software tools. Annotation was performed using the Plann (Huang and Cronk 2015), then a physical map of the chloroplast genome generated by Genome Vx (Conant and Wolfe 2008).

The cp genome of *C. bretschneideri* was 159,607 bp in length, and consisted of a large single-copy (LSC) region (87601 bp), a small single-copy (SSC) region (19312 bp), separated by a pair of inverted repeat (IRs: 26,347 bp, each) regions. The total GC content of complete chloroplast genome, LSC, SSC, IR regions were 36.6%, 34.4%, 30.3% and 42.7%, respectively. The chloroplast DNA of *C. bretschneideri* comprised a total of 114 unique genes, including 80 protein-coding genes, 30 tRNA genes, and 4 rRNA genes. In these genes, 19 genes were duplicated in the IR regions, 15 genes harbored a single intron, and 2 (*ycf3*, *clpP*) contained double introns.

To clarify the phylogenetic position of *C. bretschneideri*, total 31 complete chloroplast genomes were obtained from Genbank and the sister group Prunoideae was taken as an out group. All chloroplast genome sequences were aligned using MAFFT (Katoh et al. 2019), which has been deposited at doi:10.5061/dryad.qv9s4mwfg. Phylogenetic analysis was conducted using maximum-likelihood (ML) method by IQ-TREE (1.6.12) with 1000 bootstrap replicates (Nguyen et al. 2015). The phylogenetic analysis showed that *C. bretschneideri* was closely related to *C. marshallii* Eggl, rather than *C. pinnatifida* in the subfamily Maloideae (Figure 1). This suggests that *C. bretschneideri* is a distinct *Crataegus* species, rather than a variant of *C. pinnatifida.* The phylogenetic tree can provide reference for the parent selection in hawthorn breeding programme. This complete chloroplast genome can be used for future studies on genetic engineering, population and phylogeny of family Rosaceae.

**Figure 1.**
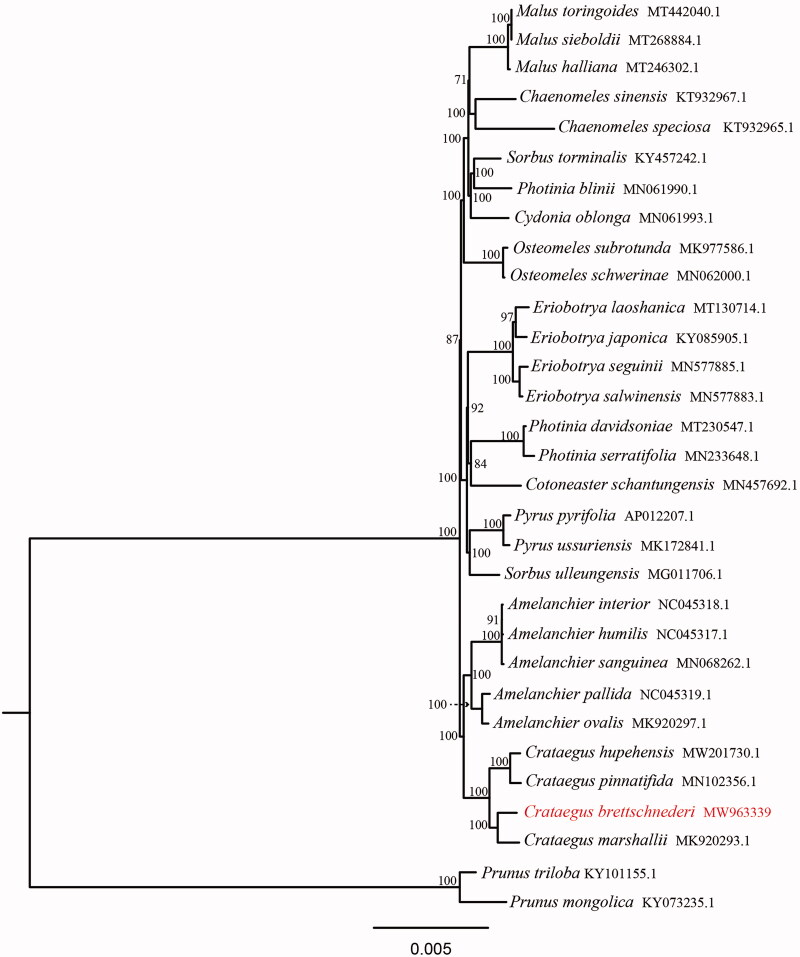
Phylogenetic tree reconstruction of 31 taxa using maximum likelihood (ML) method based on the chloroplast genome sequences. ML bootstrap support value presented at each node.

## Data Availability

The genome sequence data that support the findings of this study are openly available in GenBank of NCBI at (https://www.ncbi.nlm.nih.gov/) under the accession no. MW963339. The associated BioProject and Bio-Sample numbers are PRJNA722683, and SAMN18789926 respectively.
